# High-Sensitivity Troponin T and Incident Heart Failure in Older Men: British Regional Heart Study

**DOI:** 10.1016/j.cardfail.2018.08.002

**Published:** 2019-04

**Authors:** Paul Welsh, Olia Papacosta, Sheena Ramsay, Peter Whincup, John McMurray, Goya Wannamethee, Naveed Sattar

**Affiliations:** 1 BHF Glasgow Cardiovascular Research Centre, University of Glasgow, Glasgow, United Kingdom; 2 Department of Primary Care and Population Health, University College London, London, United Kingdom; 3 Institute of Health and Society, Newcastle University, Newcastle upon Tyne, United Kingdom; 4 Population Health Research Institute, St George's, University of London, London, United Kingdom

**Keywords:** Risk prediction, heart failure, biomarkers, troponin T

## Abstract

**Objective:**

The aim of this work was to study the association of high-sensitivity troponin T (hsTnT) with incident heart failure (HF), and implications for its use in prediction models.

**Methods and Results:**

In the British Regional Heart Study, 3852 men aged 60–79years without baseline HF (3165 without baseline chronic heart disease) were followed for a median of 12.6years, during which 295 incident cases of HF occurred (7.7%). A 1-SD increase in log-transformed hsTnT was associated with a higher risk of incident HF after adjusting for classic risk factors (hazard ratio [HR] 1.58, 95% confidence interval [CI] 1.42–1.77) and after additional adjustment for N-terminal pro–B-type natriuretic peptide (NT-proBNP; HR 1.34, 95% CI 1.19–1.52). The strength of the association between hsTnT and incident HF did not differ by strata of other risk factors. An hsTnT concentration of <5ng/L had a sensitivity of 99.7% (95% CI 98.1%–99.9%) and a specificity of 3.4% (95% CI 2.8%–4.0%). A risk-prediction model including classic risk factors and NT-proBNP yielded a C-index of 0.791, but addition of hsTnT did not further improve prediction (*P* = .28).

**Conclusions:**

Elevated hsTnT is consistently associated with risk of HF in older men. HF occurred rarely over 12years when baseline hsTnT was below the limit of detection. hsTnT measurement, however, does not improve HF prediction in a model already containing NT-proBNP.

## Highlights

• High-sensitivity troponin T is associated with 12-year risk of HF in older men.
• The association is consistent across population strata.
• The 12-year risk of HF is very low in older men with undetectable troponin T.
• Myocardial damage is therefore an important antecedent of HF.
• In a model including NT-proBNP, troponin T did not improve prediction of HF.

## Graphical abstract

**Figure:**
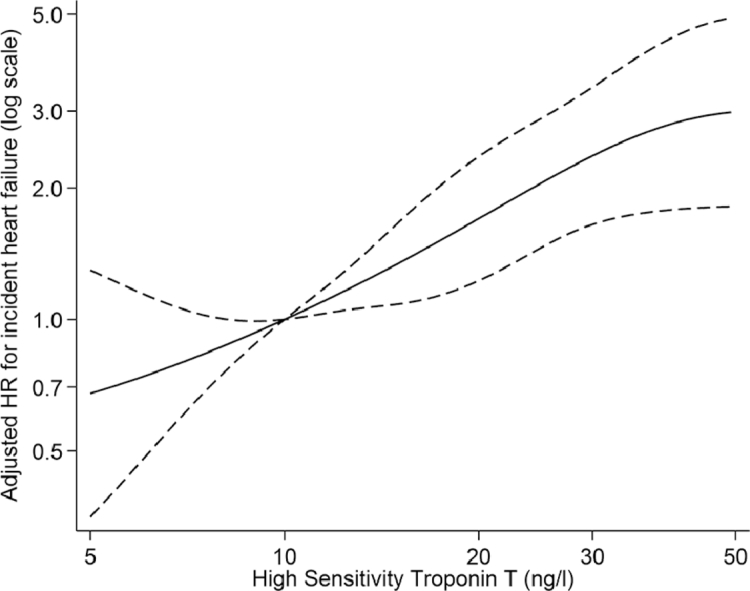

The lifetime risk of developing heart failure (HF) of a person aged >40years in the general population is estimated to be 20%, and although therapies for HF are improving, once diagnosed, 14% of patients die in the first 6 months.^1^, ^2^ The etiology of HF varies from country to country, although the majority of cases are attributed to hypertension and coronary artery disease.^3^ As such, it is well understood that onset of HF is a common occurrence after clinically diagnosed myocardial infarction.^4^ However, it is possible that recurrent subclinical episodes of cardiac ischemia and cardiomyocyte necrosis lead to HF.^5^

Circulating cardiac troponin levels are excellent biomarkers of myocardial injury, including ischemia.^6^, ^7^ As such, several studies have used high-sensitivity troponin T (hsTnT) or high-sensitivity troponin I assays as a proxy for subclinical myocardial damage in investigating causes of HF.^8^, ^9^, ^10^, ^11^, ^12^ However, it is not clear from the existing literature what information troponin measurement adds to N-terminal pro–B-type natriuretic peptide (NT proBNP). NT-proBNP integrates information about cardiac loading, structure, and function, the heart rhythm, renal function, and possibly other neurohumoral pathways. Because natriuretic peptides are increasingly part of the clinical definition of HF^13^ and are routinely measured during the diagnostic evaluation of patients with suspected HF, NT-proBNP is a strong candidate biomarker for prediction models in people without HF. It is also possible that troponin will also be valuable in such predictive models. A key question is not only whether continuous troponin levels predict HF, but also whether low troponin levels, indicating an absence of myocardial injury, preclude occurrence of HF.

We therefore used the British Regional Heart study to test the hypothesis that hsTnT is associated with risk of incident HF in older men with and without clinical evidence of baseline coronary heart disease (CHD), adjusting for incident CHD during follow-up. We also examined whether the measurement of hsTnT usefully predicts risk of incident HF beyond the measurement of NT-proBNP.

## Methods

### British Regional Heart Study

The British Regional Heart Study was a socioeconomically representative prospective study of 7735 men aged 40–59years and of predominantly white European ethnicity (>99%), drawn from 1 general practice in each of 24 British towns, who were screened from 1978 to 1980.^14^ In 1998–2000, surviving men aged 60–79years were invited for a 20th-year follow-up examination, on which the present analyses were based.^15^, ^16^ Ethical approval was obtained from all relevant local Research Ethics Committees, and informed consent had been obtained from the subjects. Follow-up was possible for 99% of the cohort. All of the men completed a mailed questionnaire providing information on their lifestyle and medical history, had a physical examination, and provided a fasting blood sample. Physical activity, alcohol consumption, and an index of socioeconomic deprivation were derived and coded as detailed elsewhere.^17^ Twelve-lead electrocardiograms were recorded with the use of a Siemens Sicard 460 instrument and analyzed according to Minnesota Coding definitions at the University of Glasgow electrocardiography core laboratory. Atrial fibrillation was defined according to Minnesota Codes 8.3.1 and 8.3.3 on the baseline electrocardiography. The men were asked whether a doctor had ever told them that they had myocardial infarction (MI), HF, or stroke; details of their medications, including use of statins, were recorded at the examination. Predicted glomerular filtration rate (eGFR) was estimated from serum creatinine: eGFR = 186 × (creatinine)^−1.154^ × (age)^−0.203^.

In all, 4252 men (77% of the survivors) attended the 1998–2000 examination; 130 men who experienced HF before the baseline examination were excluded; 79 men with missing information on history of MI or angina were also excluded; and an additional 191 with a missing hsTnT measurements were excluded, leaving 3852 men included in the analysis.

Baseline CHD was defined as previous self-reported, doctor-diagnosed, MI or angina, or MI identified during review of medical records as part of the prospective study; in this way, 687 men were defined as having evidence of CHD at baseline.

Evidence of HF was obtained by reports from primary care physicians supplemented by biennial reviews of medical records (including correspondence). Incident HF was based on a confirmed doctor’s diagnosis of HF from primary care records and where possible, verified using details of available clinical information from primary and secondary care records, as well as from death certificates (ICD-9 code 428).^16^

### Biomarker Measurement

NT-proBNP and hsTnT were measured in plasma samples from both studies on an automated clinically validated immunoassay analyzer (e411; Roche Diagnostics, Burgess Hill, United Kingdom) using the manufacturers calibrators and quality control reagents. The limit of detection was 5ng/L for NT-proBNP, and the limit of blank was 3ng/L for hsTnT. We defined “low” hsTnT as the manufacturer's limit of detection (5 ng/L). Quality control materials over 2 levels for each biomarker ran from 4.4% to 7.7%.

### Statistics

Eligible men were divided into equal tertiles based on the hsTnT distribution. Skewed continuous variables were log-transformed to approximate normality for parametric tests. Comparisons of baseline characteristics between the HF outcome groups (stratified by baseline CHD status) were performed with the use of the χ^2^ test for categoric variables and *t* test for continuous variables; 95% confidence intervals for single proportions were derived with the use of the Jeffrey method.

Kaplan-Meier curves and the log-rank test were used to evaluate differences in HF rates for the hsTnT tertiles. Multiple imputation with the use of chained equations was used to generate 10 datasets with complete data.^18^ The imputation model included hsTnT, age, cardiovascular disease (CVD), diabetes, incident HF (none missing), smoking (4 missing), index of deprivation (6 missing), atrial fibrillation (9 missing), heart rate (10 missing), systolic blood pressure (17 missing), body mass index (BMI; 17 missing), C-reactive protein (CRP; 31 missing), eGFR (35 missing), total cholesterol (38 missing), glucose (39 missing), forced expiratory volume in 1 second (FEV_1_; 40 missing), blood pressure medications (49 missing), alcohol use (57 missing), high-density lipoprotein (HDL) cholesterol (61 missing), physical activity (138 missing), and NT-proBNP (272 missing). Cox proportional hazards models were generated using the “mi estimate” command in Stata. The hazard ratio (HR) and 95% confidence intervals (CIs) of incident HF per a 1-SD increase in log-transformed hsTnT was estimated with the use of these models. Models adjusted for classic risk factors, with the maximally adjusted model including the variables age, smoking, total cholesterol, HDL cholesterol, systolic blood pressure, index of multiple deprivation (IMD), BMI, any diabetes, eGFR, blood pressure medication, statin use, heart rate, physical activity, FEV_1_, alcohol consumption, CRP, and NT-proBNP. Models also included a time-varying term adjusting for nonfatal MI that occurred during the follow-up. Maximally adjusted models were also tested for evidence of interaction, stratified by baseline covariates of interest. The assumption of a linear relationship between hsTnT and risk of HF was supported by restricted cubic spline analysis.

To test clinical prediction of incident HF, C-indexes were calculated in the imputed datasets, accounting for censoring, with the use of a range of different prediction strategies (with or without hsTnT). Prediction models included model A) age, baseline CHD, cholesterol, HDL cholesterol, systolic blood pressure, IMD, BMI, smoking, diabetes, eGFR, blood pressure medication, statin use, heart rate, glucose, physical activity, FEV_1_, alcohol use, atrial fibrillation; model B) model A plus NT-proBNP; model C) model B plus hsTnT; and model D) age and NT-proBNP only. Increased concordance was tested by comparing the C-index of each model with every other one.

The C-statistic has been criticized for insensitivities to changes in clinical decisions across treatment thresholds defined by risk prediction. The categoric net reclassification index (NRI) estimates correct changes in clinical classification across risk thresholds. However, risk score thresholds are currently not used to make treatment decisions to prevent HF. Therefore, we used a continuous net reclassification index (NRI), to compare model C with model B in one imputed dataset. NRI is based on improvements in classification across integer-percentage risk thresholds, thus avoids making arbitrary decisions about defining clinically relevant risk categories.^19^ We also calculated the integrated discrimination improvement index (IDI), a category-free comparative measure of the clinical validity of a new risk score.^20^

All analyses were performed in Stata (version 14.2) and R (version 3.3.1, with the use of the survIDINRI package and 5000 bootstrap samples to generate confidence intervals for the NRI and IDI).

## Results

### Classic Risk Factors

During a median follow-up period of 12.6years (interquartile range [IQR] 7.9–13.4 y), there were 295 incident cases of HF in the whole cohort (n = 3852; incidence 7.7%), including 201 incident cases (6.4%) in those without baseline CHD (n = 3165) and 94 incident cases (13.7%) in those with baseline CHD (n = 687; *P* < .001). Those who experienced HF during follow-up were generally older, had higher BMI and waist circumference, were more likely to have diabetes, had lower FEV_1_ and eGFR, had higher heart rate, were more likely to be on blood pressure–lowering medication and to have atrial fibrillation and higher CRP (Table1). hsTnT was slightly higher in those with baseline CHD compared with those without: median 12.9 ng/L (IQR 9.9–18.2) vs 11.4ng/L (8.6–15.6); *P* < .001. NT-proBNP was also higher in those with clinical evidence of baseline CHD compared to those without: median 180ng/L (88–416) vs 79ng/L (41–158); *P* < .001.

**Table1:** Distribution of Risk Factors at Baseline Comparing Those Who Experienced HF During Follow-Up With Those Who Did Not

Risk Factor	No Incident HF (n = 3557)	Incident HF (n = 295)	*P* Value
Age, y	68.5 (5.5)	70.5 (5.4)	.001
BMI, kg/m^2^	26.8 (3.6)	27.8 (3.9)	.001
Waist circumference, cm	96.7 (10.4)	99.7 (11.2)	.001
Smoking			.42
Never	1048 (29.5%)	79 (26.8%)	
Ex	2039 (57.4%)	181 (61.4%)	
Current	466 (13.1%)	35 (11.9%)	
FEV_1_, L	2.62 (0.65)	2.40 (0.68)	.001
SBP, mmHg	149.1 (24.0)	152.2 (25.2)	.04
DBP, mmHg	85.4 (11.1)	84.8 (11.0)	.43
Heart rate, beats/min	65.5 (12.5)	67.6 (13.7)	.006
Total cholesterol, mmol/L	6.02 (1.07)	5.90 (1.12)	.08
HDL cholesterol, mmol/L	1.32 (0.34)	1.29 (0.34)	.14
Glucose, mmol/L	5.99 (1.79)	6.30 (2.55)	.006
Physical activity			*.*03
Inactive	360 (10.5%)	35 (12.3%)	
Occasional–light	1424 (41.5%)	127 (44.6%)	
Moderate–vigorous	1645 (48.0%)	123 (43.2%)	
Alcohol			*.*79
None	347 (9.9%)	32 (11.2%)	
Occasional–light	2469 (70.5%)	205 (69.7%)	
Moderate–heavy	688 (19.6%)	54 (19.1%)	
Diabetes	232 (6.5%)	33 (11.2%)	*.*002
Atrial fibrillation	105 (3.0%)	30 (10.2%)	*<.*001
Statin	212 (6.0%)	25 (8.5%)	*.*084
Blood pressure medication	1056 (30.1%)	139 (47.8%)	*<.*001
IMD, score	20.2 (14.7)	20.6 (14.7)	*.*66
eGFR, ml/min/1.73m^2^	72.8 (12.6)	70.1 (13.4)	*<.*001
CRP, mg/L	1.54 (0.81–3.33)	2.16 (1.04–4.18)	*<.*001
NT-proBNP, pg/ml	85 (44–173)	231 (102–577)	*<.*001
hsTnT, ng/L	11.5 (8.7–15.6)	15.5 (11.0–20.1)	*<.*001

Values are presented as mean (SD), n (%), or median (interquartile range).

BMI, body mass index; CRP, C-reactive protein; DBP, diastolic blood pressure; eGFR, estimated glomerular filtration rate; FEV_1_, forced expiratory volume in 1 second; HDL, high-density lipoprotein; HF, heart failure; hsTnT, high-sensitivity troponin T; IMD, index of multiple deprivation; NT-proBNP, N-terminal pro–B-type natriuretic peptide; SBP, systolic blood pressure.

### hsTnT and Incident HF

**Fig.1:**
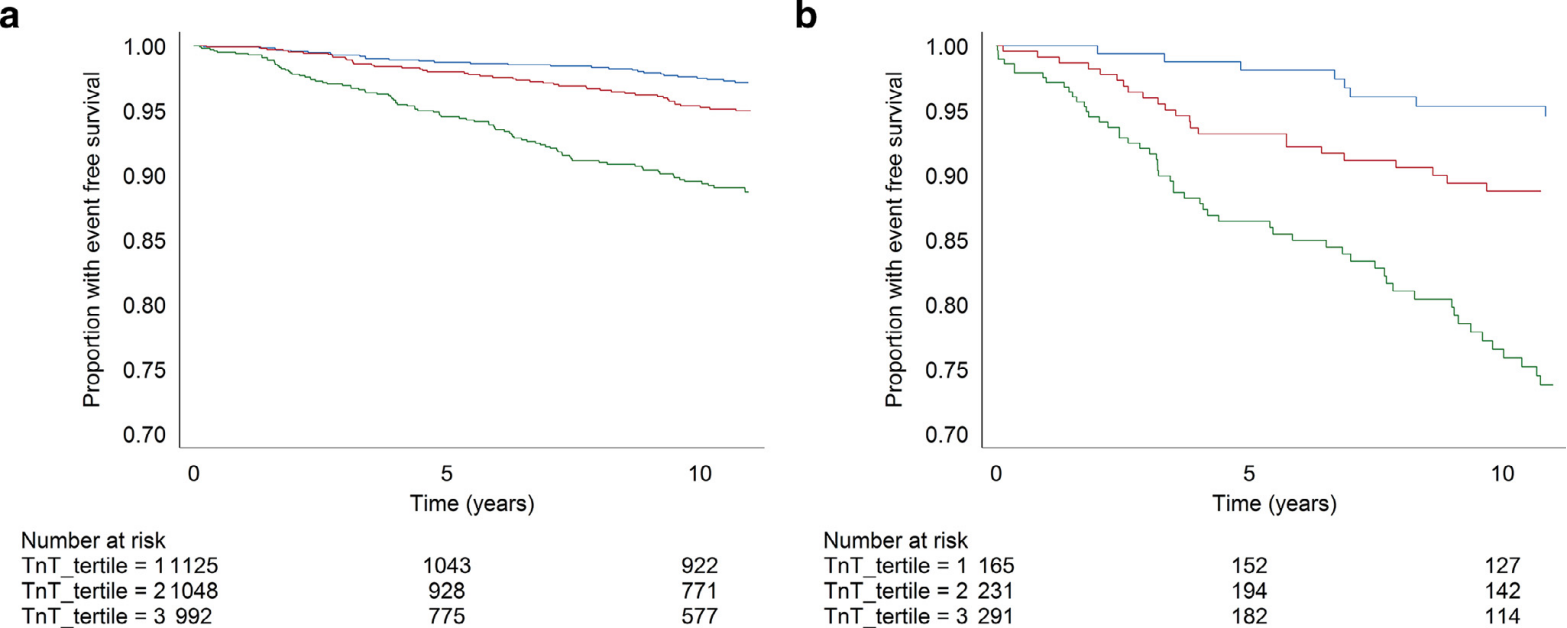
Kaplan-Meier curves illustrating heart failure (HF)–free survival by tertiles of hsTnT in (a) those without and (b) those with baseline coronary heart disease. Blue curves are the lowest tertile (≤9.7 ng/L), red the middle tertile (9.8–14.2 ng/L), and green the highest tertile (≥14.3 ng/L). Cutoffs are defined from the whole cohort.

Kaplan-Meier curves show that over the follow-up time, baseline hsTnT concentration (as thirds of the distribution) was strongly associated with risk of incident HF in both those with and without baseline CHD (log rank *P* < .001 for both; Fig.1).

**Fig.2:**
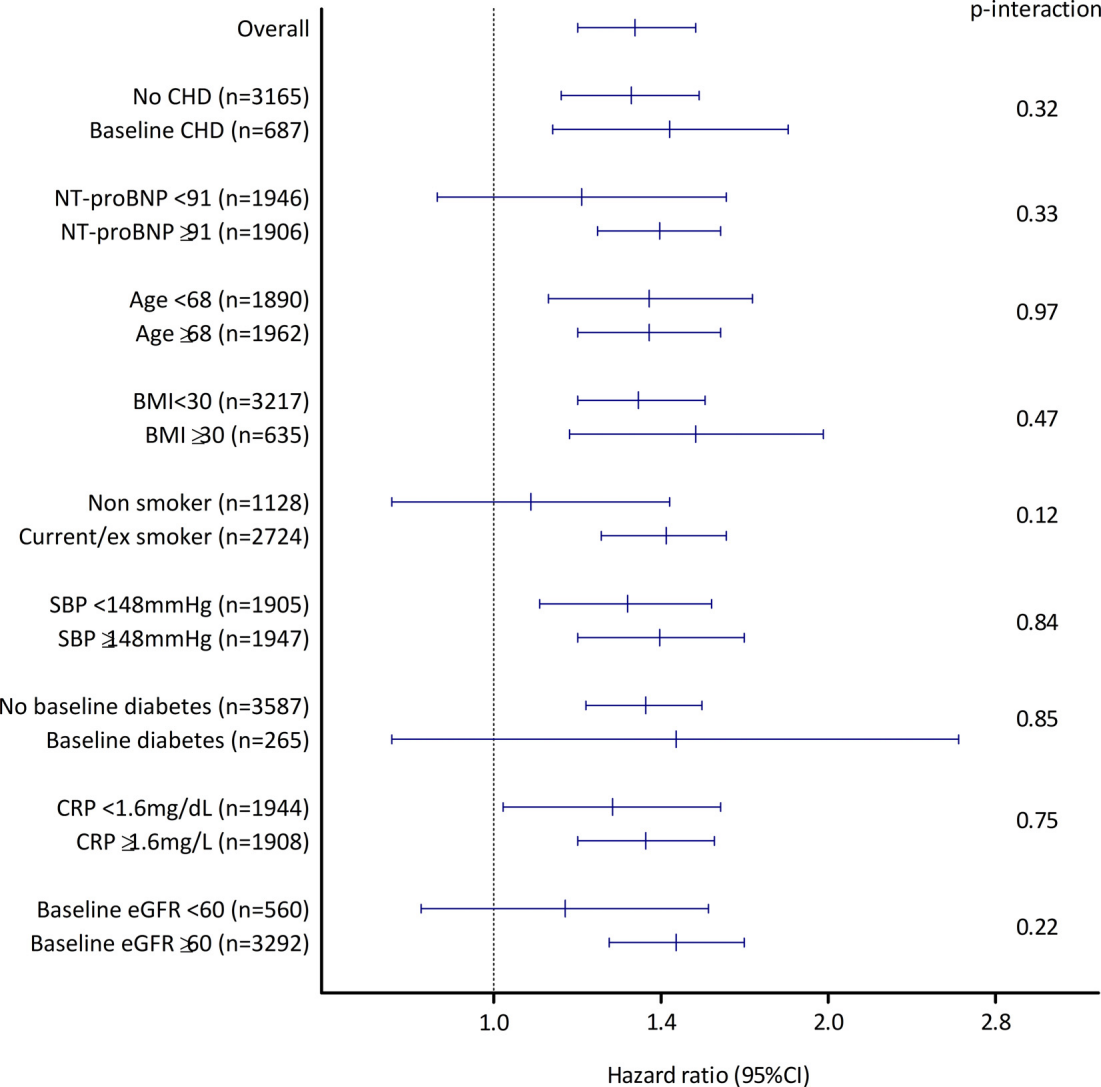
Hazard ratios (with 95% confidence intervals [CIs]) for heart failure per 1 SD increase in hsTnT, stratified by a range of other risk factors. *P* values are tests for interaction comparing the effect of hsTnT in stratified groups. CHD, coronary heart disease; NT-proBNP, N-terminal pro–B-type natriuretic peptide; BMI, body mass index; SBP, systolic blood pressure; CRP, C-reactive protein; eGFR, estimated glomerular filtration rate.

In adjusted models, the association of log-transformed hsTnT with HF was linear. In all participants, a 1-SD higher log-hsTnT was associated with a 58% (95% CI 42%–77%) higher risk of HF after adjusting for classic risk factors (model 3; Table2), and this was attenuated to a 34% higher risk (95% CI 19%–52%) after adjusting for NT-proBNP as well (model 4). A test for interaction between the risk of HF associated with baseline hsTnT concentration and baseline CHD status was not significant: *P* = .32; Fig 2). Indeed, there was no evidence that level of any risk factor modified the association between hsTnT and risk of HF (Fig 2).

**Table2:** Associations of hsTnT (Per SD Increase on Log Scale) With Heart Failure

Study/Events	N (n events)	Model 1		Model 2		Model 3		Model 4	
		HR (95% CI)	*P* Value	HR (95% CI)	*P* Value	HR (95% CI)	*P* Value	HR (95% CI)	*P* Value
All participants	3852 (295)	1.81 (1.66–1.97)	<.001	1.64 (1.47–1.82)	<.001	1.58 (1.42–1.77)	<.001	1.34 (1.19–1.52)	<.001
Participants without baseline CHD	3165 (201)	1.76 (1.57–1.96)	<.001	1.59 (1.40–1.81)	<.001	1.51 (1.32–1.74)	<.001	1.33 (1.15–1.53)	<.001
Participants with baseline CHD	687 (94)	1.82 (1.55–2.12)	<.001	1.79 (1.47–2.18)	<.001	1.83 (1.48–2.26)	<.001	1.44 (1.13–1.84)	.001

Model 1: unadjusted. Model 2: adjusted for age, total cholesterol, HDL cholesterol, systolic blood pressure, IMD, BMI, smoking, diabetes, eGFR, blood pressure medication use, statin use, and myocardial infarction that occurred during follow-up. Model 3: additionally adjusted for heart rate, glucose, physical activity, FEV_1_, alcohol use, atrial fibrillation, and CRP. Model 4: additionally adjusted for NT-proBNP. CHD, coronary heart disease; CI, confidence interval; HR, hazard ratio; other abbreviations as in Table1.

### Prediction of HF

Of the 121 men with a baseline hsTnT <5ng/L (the assay limit of detection), only 1 developed new-onset HF (0.8%, 95% CI 0%–3.8%), compared with 294 of the 3731 with detectable hsTnT (7.9%, 95% CI 7.1%–8.8%). This corresponds to a sensitivity of 99.7% (95% CI 98.1%–99.9%) and a specificity of 3.4% (95% CI 2.8%–4.0%). Only 35 participants had an NT-proBNP below the limit of detection (5 pg/mL), and none of those developed HF during follow-up.

An HF risk score based on classic risk factors (without sex or race) yielded a C-index of 0.730 (model A; Table3). Addition of NT-proBNP improved the C-index substantially to 0.791 (model B). However, further addition of hsTnT (model C) did not further improve prediction (C-index 0.794). A model based on age and NT-proBNP only (model D) yielded a C-index of 0.757 and was almost as good at discriminating HF events as any of the other, more complex, models.

**Table3:** Prediction of Heart Failure in Those Without Baseline Coronary Heart Disease: C-Statistics Matrix Comparing Various Models With and Without hsTnT (n = 3852)

Model	C-index	vs Model A	vs Model B	vs Model C
Model A: classic risk factors*	0.730 (0.701–0.759)			
Model B: classic risk factors* plus NT-proBNP	0.791 (0.766–0.816)	+0.061 (0.039–0.082) *P < .*001		
Model C: classic risk factors* plus NT-proBNP and hsTnT	0.794 (0.769–0.819)	+0.064 (0.043–0.086) *P < .*001	+0.004 (−0.003 to 0.010) *P = .*28	
Model D: age and NT-proBNP	0.757 (0.729–0.785)	+0.027 (−0.004 to 0.058) *P = .*09	−0.034 (−0.050 to −0.018) *P < .*001	−0.037 (−0.055 to −0.020) *P < .*001

Abbreviations as in Table1.

⁎ Classic risk factors: Age, total cholesterol, HDL cholesterol, systolic blood pressure, IMD, BMI, smoking, diabetes, eGFR, blood pressure medication use, statin use, heart rate, glucose, physical activity, FEV_1_, alcohol use, atrial fibrillation, and baseline cardiovascular disease.

With the use of other prediction metrics, adding hsTnT to a model containing NT-proBNP (models C vs B) did not improve the continuous NRI (+6.7%, 95% CI −4.9 to 16.0%; *P* = .26), although there was a slight improvement in the IDI (+0.013, 95% CI 0.003–0.026; *P* = .006). Data were similar when stratified by baseline CHD (data not presented).

## Discussion

In this cohort of older British men, hsTnT was consistently strongly associated with incident HF, suggesting that subclinical myocardial damage may be an important risk factor for HF, even in individuals without diagnosed CHD. HF rarely occurred during 12.6years of follow-up (0.8% incidence, 95% CI 0%–3.8%) in men who had a baseline hsTnT below the limit of detection (5 ng/L). As such, these data raise mechanistic questions regarding the source of troponin elevation in apparently healthy men, and how pathologies that raise troponin might be associated with increased HF risk. Although useful in predicting HF when added to conventional risk factors, TnT did not improve HF prediction when added to NT-proBNP.

### HF Prediction

One of the reasons NT-proBNP is such an attractive and powerful biomarker in CVD prediction in general^17^, ^21^^,^^22^ is that it integrates information from several important pathophysiologic pathways. As well as reflecting cardiac overload, NT-proBNP is a marker of myocardial ischemia, therefore overlapping with troponin.^23^, ^24^ Circulating levels of both NT-proBNP and hsTnT probably also reflect renal function.^25^ Despite this, we still found a moderate association between hsTnT and HF, even after adjusting for classic risk factors and NT-proBNP. However, once NT-proBNP is included in a model for HF prediction, the baseline C-statistic is already high (0.79) and is therefore difficult to improve upon. Data from the ARIC (Atherosclerosis Risk in Communities) study showed that when TnT was added to a range of classic risk factors plus NT-proBNP, the C-index for HF prediction was improved statistically significantly, but only clinically modestly (by +0.014 in men and +0.012 in women).^9^ Although the present study is smaller in size, our data broadly agree with those of ARIC: if hsTnT does predict HF beyond NT-proBNP, the discrimination gained is very moderate. Our data also advance findings from studies of patients with established acute and chronic HF. For example, in chronic HF patients in the RED-HF (Reduction of Events by Darbepoetin Alfa in Heart Failure) trial, among a range of cardiac biomarkers only hsTnT added to NT-proBNP in the prediction of adverse outcome.^26^ In the MARATHON (Multicentre Australian Risk Algorithm to Predict Heart Failure Readmission) study, comprising patients hospitalized with preserved or reduced ejection fraction HF, elevated troponin I predicted 30-day readmission to hospital or death, both individually and as part of a panel of variables.^27^ Therefore, elevated troponin appears to be a predictor of adverse outcomes in the presymptomatic, chronic, and acute phases of HF.

### Mechanisms Linking Troponin and HF

In line with these being older men, the median baseline hsTnT level in this cohort was ∼12ng/L (the 99th percentile in the healthy population is 14 ng/L) and was only slightly higher in those with clinically identified baseline CHD. CHD is the most obvious cause of elevated troponin, although only a minority of our participants had diagnosed CHD at baseline. However, even in the absence of a diagnosis of CHD, the prevalence of subclinical coronary disease was probably high in the older British men we studied. In addition, other potentially undiagnosed cardiac conditions, such as left ventricular hypertrophy and atrial fibrillation, may cause myocardial ischemia. Hypertension, diabetes, and chronic kidney disease (which frequently coexist) lead to the development of left ventricular hypertrophy and atrial fibrillation, as well as coronary microvascular dysfunction, which also may raise troponin. Hypertension, diabetes, and chronic kidney disease, left ventricular hypertrophy, atrial fibrillation, and coronary microvascular dysfunction are also predictors (along with those measured in this study) and precursors of HF. Moreover, those developing HF had a higher baseline NT-proBNP concentration, suggesting preexisting myocardial and/or renal dysfunction. Other noncardiac pathologies, including chronic inflammatory disorders, also are associated with myocardial injury, and the higher baseline CRP in individuals experiencing incident HF is consistent with this pathophysiologic connection as well.^28^ Thus, there are mechanisms that might explain why troponin is associated with incident HF in those without diagnosed CHD. Many of these factors were taken into account in our statistical analysis. Future studies should investigate specifically the ability of troponin measurements to predict onset of nonischemic dilated cardiomyopathy.

It may be possible to detect subclinical myocardial injury early and to intervene to reduce the risk of HF. This might be achieved with the use of the measurement of troponin, NT-proBNP, or both to diagnose such patients and thereby target therapy to those at highest risk of HF. Interventions that might be effective include antihypertensive therapies, antiischemic therapies, lipid-lowering treatments, antithrombotic therapy, and, possibly, drugs improving metabolic status (eg, treatments for diabetes and obesity). The small but statistically significant effect of statins in reducing the risk of incident HF, independently from MI, is consistent with this hypothesis.^29^ A recent example of a hypotensive agent which also lowers troponin is sacubitril/valsartan (formerly LCZ696).^30^ Prevention of myocardial injury to reduce risk of HF in later life is therefore an important public health aim, in the same way that reduction in acute MI and stroke is.

### Study Strengths and Limitations

Strengths of the study include the prospective nature of the data and the relatively large size of the study with a long follow-up period. We used a high-quality validated clinical assay in routine use in clinical biochemistry departments to measure both NT-proBNP and hsTnT.

Weaknesses include the findings being based on doctor-diagnosed HF, and although diagnoses were usually supported by evidence from hospital investigations, there is likely to be some outcome misclassification, including underreporting of incident HF. Because we did not have echocardiographic data, we were unable to differentiate incident HF with reduced ejection fraction from HF with preserved ejection fraction. Similarly, routine coronary angiography was not performed, in common with most cohort studies. These limitations mean that we can not be sure to what extent information gained from measuring hsTnT overlaps with information that have might been available had other diagnostic investigations been performed. However, a potential use of cardiac biomarkers is to select the most appropriate patients in which to carry out these investigations.^31^, ^32^ Finally, we studied older male survivors from a socioeconomically representative general cohort study, but participants were a predominantly white population of European origin, so that the results can not be generalized directly to women, younger individuals, or other ethnic groups. However, additional studies, such as ARIC (ages 45–64 y at baseline),^9^ also demonstrated an association of hsTnT with incident HF in both men and women in an ethnically diverse cohort, providing external validity.

## Conclusions

Although NT-proBNP is the most powerful predictor of HF, even low-grade elevation of hsTnT is consistently associated with a higher risk of HF in older men, and HF rarely occurs when hsTnT is near the limit of detection. Interventions to prevent myocardial injury may mitigate risk of HF in later life.

## Disclosures

P.W. has received grant support from Roche Diagnostics. N.S. reports consulting, speaking, and/or honoraria from Amgen, Roche Diagnostics, Sanofi/Regeneron, Astra Zeneca, Boehringer Ingelheim, Novo Nordisk, and Janssen. J.M. reports consulting fees from Cytokinetics/Amgen. All of the other authors report no potential conflict of interest.
